# Oxygen isotopes in orangutan teeth reveal recent and ancient climate variation

**DOI:** 10.7554/eLife.90217

**Published:** 2024-03-08

**Authors:** Tanya M Smith, Manish Arora, Christine Austin, Janaína Nunes Ávila, Mathieu Duval, Tze Tshen Lim, Philip J Piper, Petra Vaiglova, John de Vos, Ian S Williams, Jian-xin Zhao, Daniel R Green

**Affiliations:** 1 Griffith Centre for Social and Cultural Research, Griffith University Southport Australia; 2 Australian Research Centre for Human Evolution, Griffith University Southport Australia; 3 Department of Environmental Medicine and Public Health, Icahn School of Medicine at Mount Sinai New York United States; 4 School of the Environment, The University of Queensland Brisbane Australia; 5 Centro Nacional de Investigación sobre la Evolución Humana (CENIEH) Burgos Spain; 6 Palaeoscience Labs, Department of Archaeology and History, La Trobe University Melbourne Australia; 7 Department of Geology, Universiti Malaya Kuala Lumpur Malaysia; 8 School of Archaeology and Anthropology, The Australian National University Canberra Australia; 9 Department of Geology, Naturalis Biodiversity Center Leiden Netherlands; 10 Research School of Earth Sciences, The Australian National University Canberra Australia; 11 Radiogenic Isotope Facility, School of the Environment, The University of Queensland Brisbane Australia; 12 Department of Human Evolutionary Biology, Harvard University Cambridge United States; University of California, Merced United States; Pennsylvania State University United States

**Keywords:** orangutan, *Pongo abelii*, *Pongo pygmaeus*, paleoenvironment, Other

## Abstract

Studies of climate variation commonly rely on chemical and isotopic changes recorded in sequentially produced growth layers, such as in corals, shells, and tree rings, as well as in accretionary deposits—ice and sediment cores, and speleothems. Oxygen isotopic compositions (δ^18^O) of tooth enamel are a direct method of reconstructing environmental variation experienced by an individual animal. Here, we utilize long-forming orangutan dentitions (*Pongo* spp.) to probe recent and ancient rainfall trends on a weekly basis over ~3–11 years per individual. We first demonstrate the lack of any consistent isotopic enrichment effect during exclusive nursing, supporting the use of primate first molar teeth as environmental proxies. Comparisons of δ^18^O values (n=2016) in twelve molars from six modern Bornean and Sumatran orangutans reveal a high degree of overlap, with more consistent annual and bimodal rainfall patterns in the Sumatran individuals. Comparisons with fossil orangutan δ^18^O values (n=955 measurements from six molars) reveal similarities between modern and late Pleistocene fossil Sumatran individuals, but differences between modern and late Pleistocene/early Holocene Bornean orangutans. These suggest drier and more open environments with reduced monsoon intensity during this earlier period in northern Borneo, consistent with other Niah Caves studies and long-term speleothem δ^18^O records in the broader region. This approach can be extended to test hypotheses about the paleoenvironments that early humans encountered in southeast Asia.

## eLife digest

When an animal drinks water, two naturally occurring variants of oxygen – known as oxygen-18 and oxygen-16 – are incorporated into its growing teeth. The ratio of these variants in water changes with temperature, rainfall and other environmental conditions and therefore can provide a record of the climate during an animal’s life. Teeth tend to be well preserved as fossils, which makes it possible to gain insights into this climate record even millions of years after an animal’s death.

Orangutans are highly endangered great apes that today live in rainforests on the islands of Borneo and Sumatra. During a period of time known as the Pleistocene (around 2.6 million years to 12,000 years ago), these apes were more widely spread across Southeast Asia. Climate records from this area in the time before human-induced climate change are somewhat limited. Therefore, fossilized orangutan teeth offer a possible way to investigate past seasonal rainfall patterns and gain insight into the kind of environments early humans would have encountered.

To address this question, Smith et al. measured oxygen-18 and oxygen-16 variants in thin slices of modern-day orangutan teeth using a specialized analytical system. This established that the teeth showed seasonal patterns consistent with recent rainfall trends, and that the ratio of these oxygen variants did not appear to be impacted by milk intake in young orangutans. These findings indicated that the oxygen variants could be a useful proxy for predicting prehistoric weather patterns from orangutan teeth.

Further measurements of teeth from fossilized Sumatran orangutans showed broadly similar rainfall patterns to those of teeth from modern-day orangutans. On the other hand, fossilized teeth from Borneo suggested that the environment used to be drier, with less intense wet seasons.

The approach developed by Smith et al. provides an opportunity for scientists to leverage new fossil discoveries as well as existing collections to investigate past environments. This could allow future research into how climate variation may have influenced the spread of early humans through the region, as well as the evolution of orangutans and other endangered animals.

## Introduction

Present-day rainfall patterns in Indonesia are controlled by the Asian and Australian monsoon systems, yielding annual trends that vary considerably with geography, topography, and the direction of monsoonal winds (Aldrian and Dwi Susanto, 2003; Moron et al., 2009; Qian et al., 2013; Belgaman et al., 2017). Northern Sumatra and western Borneo experience high annual rainfall and relatively stable annual temperatures, with a bimodal distribution of rainfall governed by the Intertropical Convergence Zone (Van Schaik, 1986; Aldrian and Dwi Susanto, 2003; Belgaman et al., 2017). These islands are also under the influence of inter-annual climate fluctuations driven by the El-Niño Southern Oscillation (ENSO); a periodic coupling of atmospheric and oceanic temperature gradients that initiates in the tropical Pacific, and influences global temperature and precipitation trends (Marshall et al., 2009).

It is well understood that variation in rainfall patterns influences the fundamental structure of primate habitats (Brockman and Van Schaik, 2005; Wessling et al., 2018). Dense tropical forests are sustained by fairly consistent rainfall and short, irregular dry seasons, while woodland communities in more arid environments have smaller trees, less dense canopies, and more deciduous trees (Vico et al., 2017; Archibald et al., 2019). In regions with prolonged dry seasons, low annual rainfall and savannah landscapes abound, in addition to disturbances such as wildfires (Pletcher et al., 2022).

Open woodland and savannah environments are unfavorable for slow-moving orangutans, the largest mammal with an arboreal lifestyle, particularly in regions with predators such as tigers or humans (Thorpe and Crompton, 2009; Ashbury et al., 2015; Spehar et al., 2018). Supra-annual ENSO events may also impact orangutan energy balance, reproduction, and social organization through the inducement of mast-fruiting, or dramatic seed production events in dipterocarp forests (Knott, 1998; Curran et al., 1999; Marshall et al., 2009). Such climate fluctuations over the past several hundred years have been documented in coral isotopes and tree-ring analyses, revealing especially marked changes during the past few decades (Cole et al., 1993; Stahle et al., 1998; Hughen et al., 1999; Urban et al., 2000; Tudhope et al., 2001; Pumijumnong et al., 2020).

Detailed climate records prior to the era of human-induced climate change are somewhat limited for island southeast Asia, but they are directly relevant to understanding the recent distribution of orangutans, and the arrival and dispersal of modern humans in the region during the Late Pleistocene (e.g. Piper, 2016; Bae et al., 2017; Spehar et al., 2018). A small number of studies of fossil corals, molluscs, marine sediments, and speleothems have provided insights into the last interglacial and glacial periods (e.g. Hughen et al., 1999; Tudhope et al., 2001; Stephens et al., 2016; Yang et al., 2016; Buckingham et al., 2022). For example, oxygen isotopes in fossil corals from seven periods during the last 130,000 years suggest that ENSO activity in the western Pacific over that time was comparable to modern records, although there was variation in the intensity of such activity at different timepoints (Tudhope et al., 2001). This study was also able to resolve bimodal annual rainfall peaks in modern corals, yet such detailed subannual records are extremely uncommon, particularly from terrestrial environments where early humans once lived alongside orangutans and other mammals.

### Oxygen isotope studies for paleoenvironmental reconstruction

Oxygen isotope values (δ^18^O) in water vary with latitude, altitude, temperature, and precipitation cycles, and are also impacted by precipitation sources. In tropical regions the primary determinant of rainfall isotope compositions is rainfall amount (Dansgaard, 1964; Rozanski et al., 1993; Belgaman et al., 2017). During wet seasons, rainfall δ^18^O values are relatively low, while the opposite pattern is evident in periods with less rain, although other meteorological factors can influence isotope values as well (Belgaman et al., 2016). This primary tropical pattern influences isotopic variation in meteoric, surface, and leaf waters, which may show further elevations in δ^18^O values during dryer periods due to preferential evaporative loss of the lighter isotope, ^16^O (da Silveira et al., 1989; Bowen, 2010; Roberts et al., 2017).

In addition to δ^18^O values in fossil corals, tree rings, and speleothems, other fine-scaled oxygen isotopic climate proxies include otoliths (fish ear bones) and mollusc shells (e.g. Aubert et al., 2012; Stephens et al., 2016; Prendergast et al., 2018)—although these are rarely preserved in rainforest environments. Records of δ^18^O values in mammalian tooth enamel are a more direct means of studying seasonality (reviewed in Green et al., 2018; Green et al., 2022), providing insight into the actual climates experienced by individuals, in contrast to indirect proxies for which it can be difficult to establish concurrence. Unlike bone, teeth do not remodel during life, and the phosphate component of the enamel mineral (hydroxyapatite) is especially resistant to modification after burial (reviewed in Smith et al., 2018a; Pederzani and Britton, 2019).

Tooth enamel is most commonly sampled with hand-held drills to recover the isotopic composition of oxygen inputs from water and food preserved in the hydroxyapatite (e.g. Janssen et al., 2016; Roberts et al., 2020; Kubat et al., 2023). This coarse drilling method yields spatially and temporally blurred powdered samples formed over a substantial and unknown period of time, however, precluding the identification of precise seasonal environmental patterns. To circumvent this limitation, we have employed the stable isotope sensitive high-resolution ion microprobe (SHRIMP SI) to measure δ^18^O values sequentially from thin sections of teeth, relating these to daily increments and birth lines to determine enamel formation times, and in some instances, calendar ages (Smith et al., 2018a; Smith et al., 2022; Green et al., 2022; Vaiglova et al., 2024).

It is well established that δ^18^O values in tooth enamel are closely related to local water oxygen isotope compositions (reviewed in Green et al., 2018; Green et al., 2022). For teeth that form after birth and during periods of milk consumption, δ^18^O values are expected to be higher, as a result of infant evaporative water loss while consuming ^18^O-enriched mother’s milk (Bryant et al., 1996; Wright and Schwarcz, 1999; Britton et al., 2015). Studies of large-bodied mammals report that milk δ^18^O values are elevated by ~1–6‰ relative to local drinking water δ^18^O (Kornexl et al., 1997; Lin et al., 2006; Chesson et al., 2010; Green et al., 2018; but see Cherney et al., 2021). Comparable data on human or nonhuman primate milk enrichment appear to be lacking, save for a study of 44 British infants aged 5–16 weeks (Roberts et al., 1988). The urine of infants who were breast-fed showed isotopic enrichment of 1–3‰ compared to infants who were fed formula prepared from sterile local tap water.

While such studies point to potential changes in infant body water during nursing, it is unclear whether such differences prohibit the use of early-formed enamel in studies of climate variation (Blumenthal et al., 2017; Luyt and Sealy, 2018). Two studies of δ^18^O values in the dentitions of modern sheep, horses, and zebras reported higher bulk values (~1–2‰) in five molars (M1) compared to the rest of the permanent dentition (Bryant et al., 1996; Fricke and O’Neil, 1996). This led Fricke and O’Neil, 1996, to suggest that M1s are unlikely to reflect the values of local meteoric water due to the influence of maternal inputs in utero and through lactation. However, near-weekly δ^18^O values over the first 2.75 years of life in a Neanderthal M1 measured with SHRIMP SI showed clear annual trends and maximum δ^18^O values corresponding to a period after nursing has ceased (Smith et al., 2018a). An examination of longer continuous periods of enamel formation within and between teeth will help to clarify whether early-formed primate teeth should be avoided for studies of climate seasonality.

**Figure 1.**
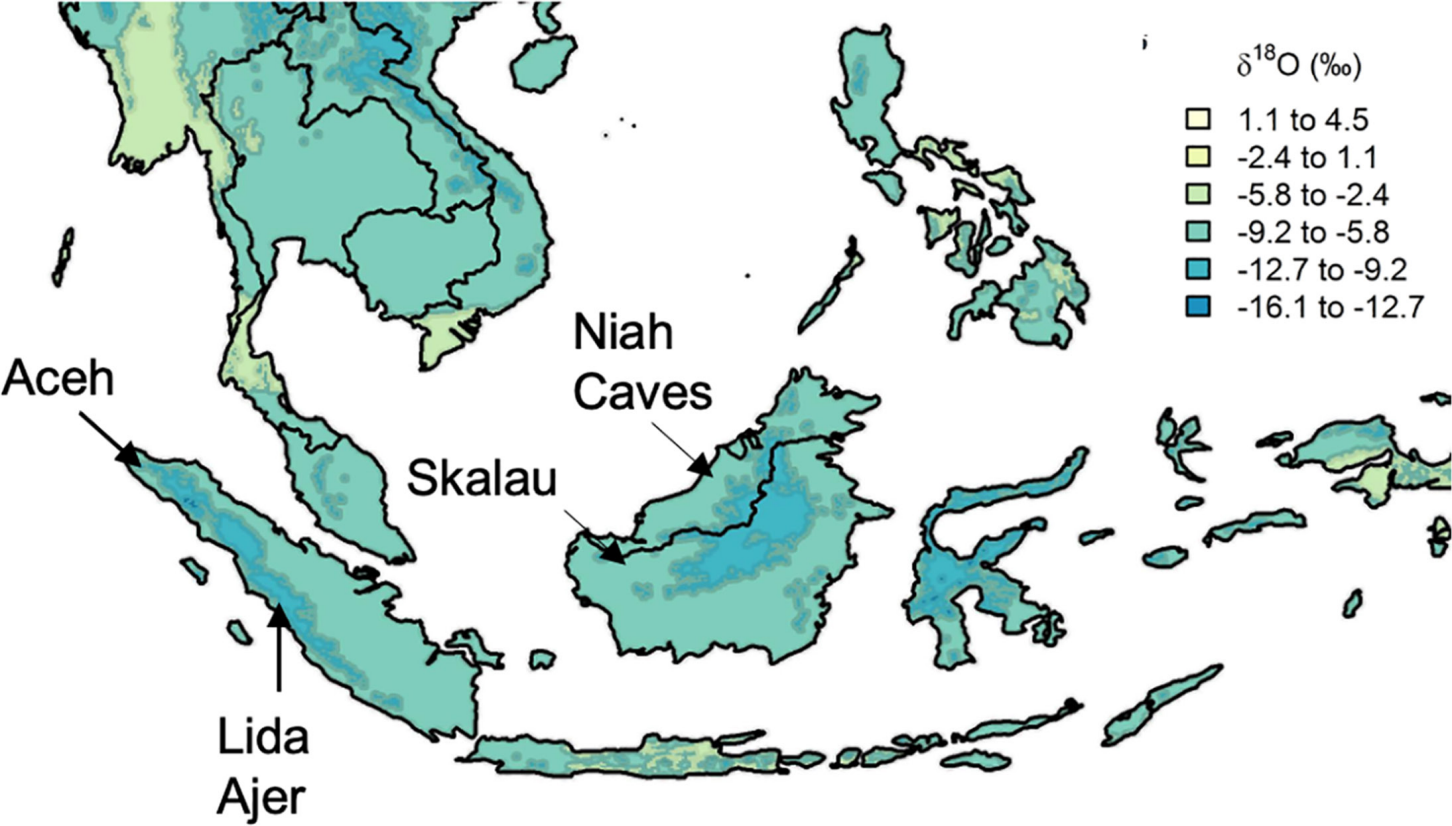
Approximate location of select modern and fossil orangutans superimposed on modeled isotopic variation. Figure modified from https://www.waterisotopes.org based on data from the Online Isotopes in Precipitation Calculator (3.0). See Table 1 for the location of particular individuals. Sibrambang Cave has yet to be relocated since Eugene Dubois’ original excavations, but it is known to be in the general vicinity of Lida Ajer in the Padang Highlands, possibly near to the modern village of a similar name (Louys et al., 2024).

Here, we first assess whether wild orangutans show elevated δ^18^O values in early-formed enamel, testing the suggestion that M1s are significantly affected by nursing ^18^O-enrichment, thereby precluding their use in climatological reconstructions. We then explore approximately 30 years of weekly δ^18^O values (n=2016 measurements) to compare orangutan individuals from the islands of Sumatra and Borneo. Finally, we contrast δ^18^O values between modern and Pleistocene orangutans, including those from key regions of early human occupation: Lida Ajer, Sumatra (Hooijer, 1948; Westaway et al., 2017) and Niah Caves, Malaysia (Hooijer, 1961; Barker et al., 2007; Figure 1, Table 1). Novel understanding of climate patterns in these fossil assemblages may inform debates about the likelihood of modern humans living in dense Asian rainforests, and the conditions that would support savannah corridors for human dispersals throughout the region (e.g. de Vos, 1983; Bird et al., 2005; Westaway et al., 2017; Louys and Roberts, 2020; Ao et al., 2024; Hamilton et al., 2024).

**Table 1.** Modern and fossil orangutan teeth employed in the current study.

Taxon	Accession	Origin	Sex	Age (years)	Teeth
*Pongo pygmaeus*	ZSM 1981/48	Skalau, Borneo	F	~8.4	RUM1, LLM2
	ZSM 1981/87	Skalau, Borneo	F	>9	LUM1, RUM2, RLM3
	MCZ 5290	Borneo (location unspecified)	n/a	4.5	RUM1
*Pongo abelii*	ZSM 1981/246	Aceh, Sumatra	M	~8.5	LLM1, LUM2
	ZSM 1981/248	Aceh, Sumatra	F	adult	LUM1, LUM2, LLM3
	ZMB 83508	Sumatra (location unspecified)	n/a	8.8	RLM1
Fossil *Pongo* spp.	11564.5	Sibrambang, Sumatra	n/a	n/a	RUM
	11565.162	Sibrambang, Sumatra	n/a	n/a	LUM
	11594.12	Lida Ajer, Sumatra	n/a	n/a	RLM
	11595.105	Lida Ajer, Sumatra	n/a	n/a	LLM
	US/22	Niah Caves, Malaysia	n/a	n/a	RLM
	Y/F4	Niah Caves, Malaysia	n/a	n/a	LLM

Numerous taxonomic assignments have been made for fossil orangutans (*Pongo* spp.), some of which have not been based on clear morphological characteristics (Tshen, 2016), and are not relevant for the focus of this paper.

## Results

### Modern orangutans

**Figure 2.**
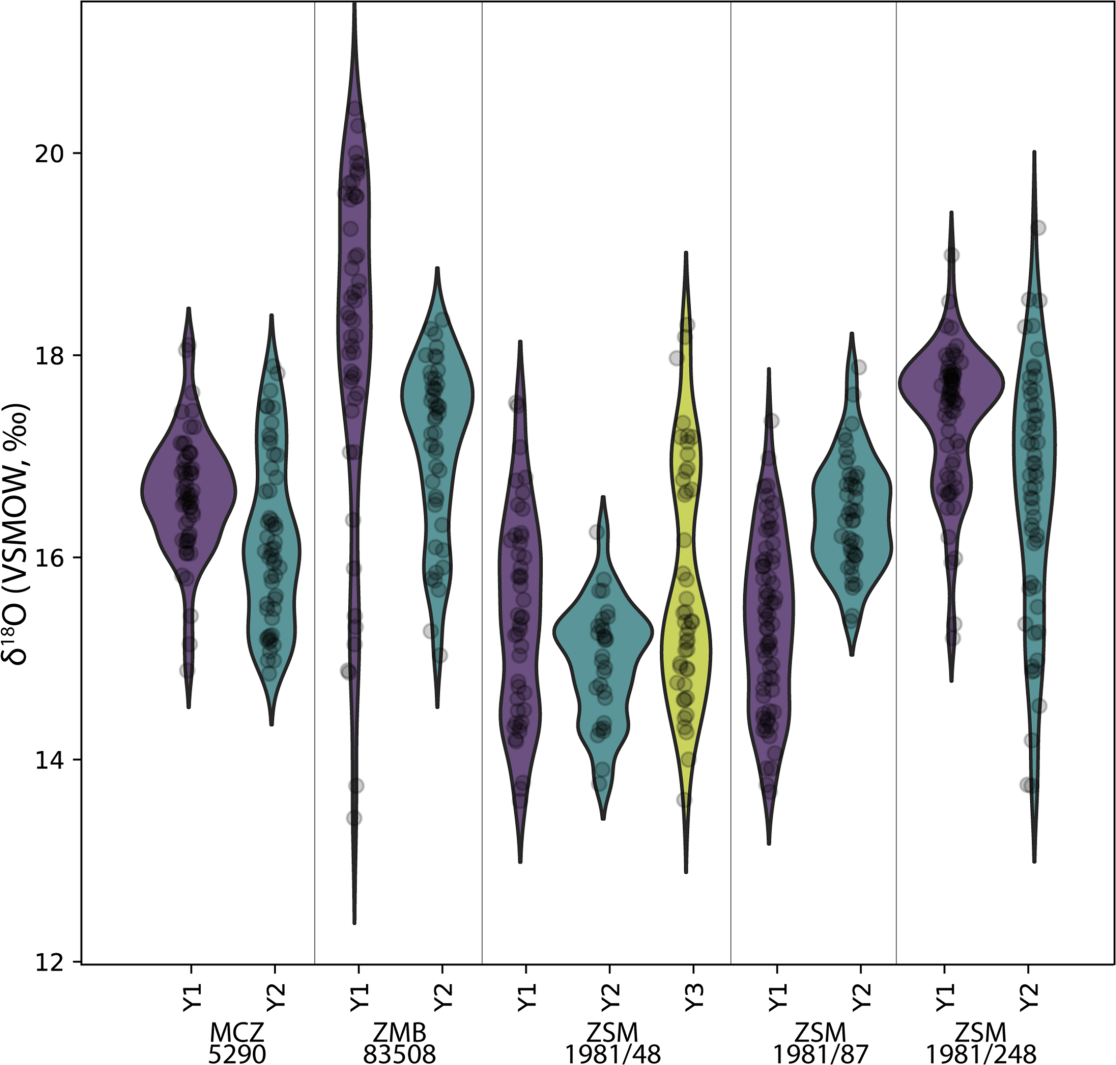
Comparison of sequential δ^18^O values across multiple years of first molar formation in five modern orangutans from Borneo and Sumatra. Bornean individuals: MCZ 5290, ZSM 1981/48, ZSM 1981/87; Sumatran individuals: ZMB 83508, ZSM 1981/248. The width of each curve is a kernel density estimate (KDE) corresponding to the distribution of δ^18^O values. First year data (Y1) is shown with a purple violin plot, second year data (Y2) with a green plot, and third year data (Y3) with a yellow plot where complete/available. Actual data are plotted as black circles.

The δ^18^O ranges of twelve modern and six fossil orangutan molars, representing 2971 near-weekly measurements spanning 57.6 years of tooth formation, are listed in Table 2. Prior to making comparisons between individuals, geographic regions, or time periods, we first consider the potential intra-individual effect of isotopic enrichment from maternal milk on δ^18^O values. Comparisons of δ^18^O values during the first, second, and third years of life in five modern orangutan first molars (M1) do not show consistently elevated values during their first year (Figure 2). Mean yearly δ^18^O values in the first year are elevated by only 0.3‰ compared to the second year. While three of the five M1s showed first year δ^18^O values higher than second year values (p≤0.05), only two individuals showed mean values that were ~1–2‰ higher during year 1; one individual showed no difference from the first to the second year, and one individual showed lower values during the first year than during the second year (p≤0.05) (Table 3). A sixth individual was only sampled from 193 days of age, but maximum values from this point onward were similar across more than 3 years of life. Similarly variable patterns were observed for the six putative fossil orangutan M1s (Appendix 1—figure 1).

**Table 2.** Modern and fossil orangutan molar δ^18^O values.

Taxon	Accession	Tooth	Cusp	Spots	Time (days)	dO18 range
*P. pygmaeus*	ZSM 1981/48	RUM1	dl	151	1241	13.6–19.9
	ZSM 1981/48	LLM2	mb	107	804	13.0–18.8
	ZSM 1981/87	LUM1	ml	131	869	13.7-17.9
	ZSM 1981/87	RUM2	ml	196	1195	12.7–20.0
	ZSM 1981/87	RLM3	mb	220	1350	13.7–19.2
	MCZ 5290	RUM1	ml	150	1002	13.8–18.1
*P. abelii*	ZSM 1981/246	LLM1	mb	136	1425	12.3–18.3
	ZSM 1981/246	LUM2	ml	229	1376	12.6–18.0
	ZSM 1981/248	LUM1	db	177	1072	11.3–19.3
	ZSM 1981/248	LUM2	db	193	1374	13.5–20.6
	ZSM 1981/248	LLM3	db	191	1461	14.8–19.6
	ZMB 83508	RLM1	db	135	1029	13.4–20.4
Fossil *Pongo* spp.	11564.5	RUM	mb	178	1387	15.3–20.4
	11565.162	LUM	ml	143	1144	14.7–20.8
	11594.12	RLM	ml	154	1081	15.1–19.9
	11595.105	LLM	mb	197	1312	15.7–20.0
	US/22	RLM	mb	149	1023	15.9–24.8
	Y/F4	LLM	db	134	869	14.2–22.9

**Table 3.** Comparisons of first and second year δ^18^O values in five first molars.

Specimen	Adjusted p-values	Higher δ^18^O values
MCZ 5290	p=0.010	Year 1
ZMB 83508	p=0.006	Year 1
ZSM 1981/48	p=0.161 (NS)	Year 1
ZSM 1981/87	p<0.001	Year 2
ZSM 1981/248	p<0.001	Year 1

**Figure 3.**
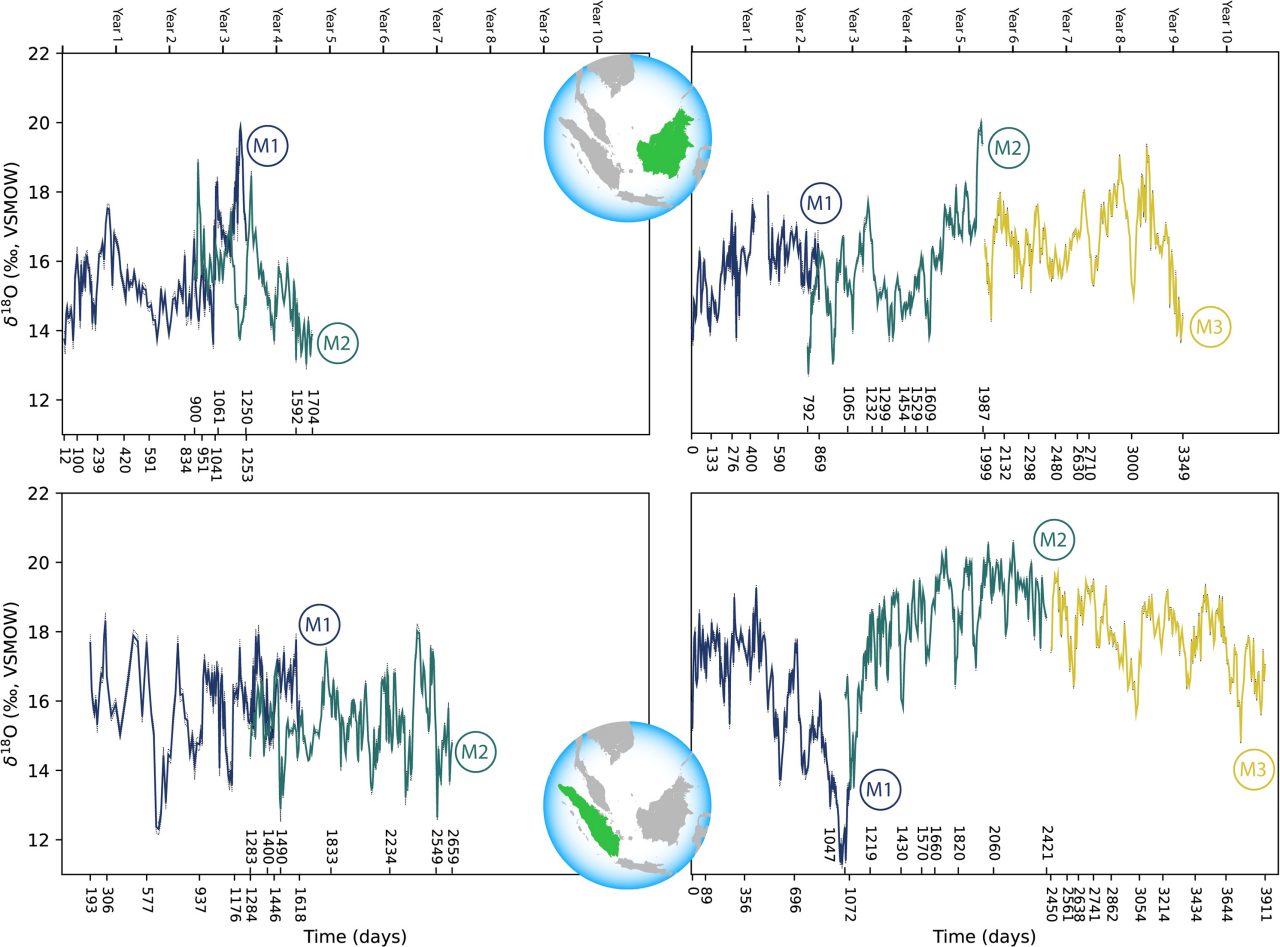
Comparison of sequential δ^18^O values across multiple years of serial molar formation in two modern orangutans from Borneo (top) and two from Sumatra (bottom). Individual in upper left: ZSM 1981/48; upper right: ZSM 1981/87; lower left: ZSM 1981/246; lower right: ZSM 1981/248. Developmental overlap was determined through registration of trace elements as in Smith et al., 2017.

Comparisons across serial molars in four modern orangutans show no consistent trend of elevated δ^18^O values in M1s relative to successive molars (Figure 3). Only two individuals showed maximum δ^18^O values in their M1s relative to M2s; in both instances M3s were unavailable due to their lack of development prior to death. The other two individuals showed higher δ^18^O values in M2s or M3s than in their respective M1s. In the case of the oldest individual (ZSM 1981/248), the highest δ^18^O values appeared at approximately 5.8 years of age, well past the age when exclusive nursing ends.

Comparison of the δ^18^O values in the full datasets of modern Bornean and Sumatran orangutans reveals a high degree of overlap. Values from the three Bornean individuals ranged from 12.7‰ to 20.0‰ (n=955 near weekly measurements), while the three Sumatran individuals ranged from 11.3‰ to 20.6‰ (n=1061 measurements). Comparisons of periodic trends via spectral power distribution analysis revealed more consistent bimodal patterns in the Sumatran individuals; three of the six Bornean molars were aperiodic (statistical power of 0.1 or less), while all six of the Sumatran molars revealed annual or semiannual cycles with greater power (Appendix 1—figure 2). Rapid oxygen isotopic shifts on the order of ~6–8‰ are evident in the single Bornean and Sumatran individuals with δ^18^O measurements spanning M1 to M3, which may represent one or more supra-annual ENSO events captured during the ~9–11 years these molars were forming.

### Fossil orangutans—oxygen isotopes

**Figure 4.**
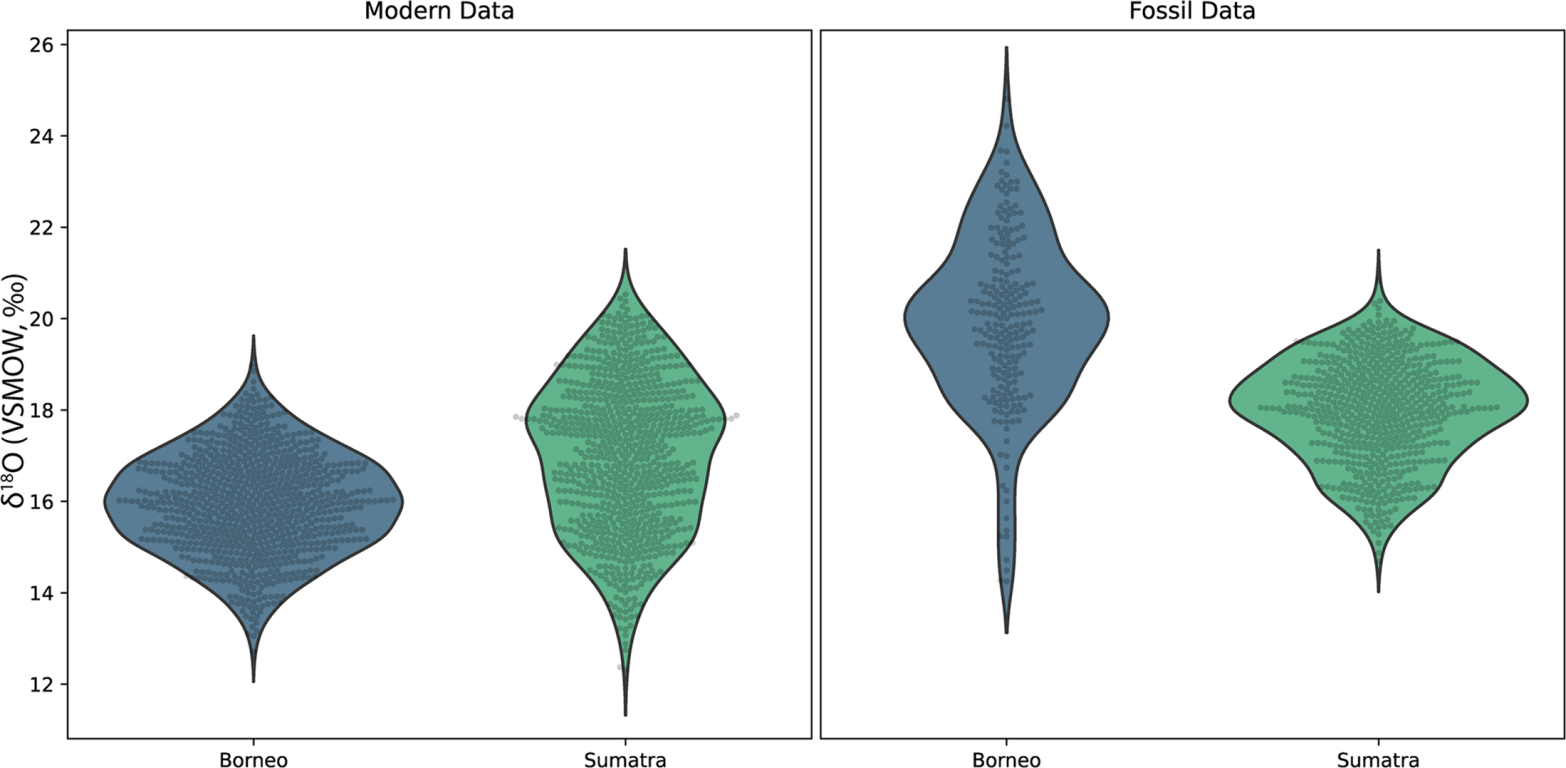
Comparison of δ^18^O values in fossil and modern orangutans from Borneo (blue) and Sumatra (green). Violin plots show kernel density estimates representing the distribution of δ^18^O values in modern individuals (left plot) and in fossil individuals (right plot). Actual δ^18^O measurements are shown as black circles.

Concurrently forming teeth (molar specimens 11594.12 and 11595.105) from same individual at Lida Ajer, Sumatra, are nearly isotopically identical; δ^18^O values range from 15.1‰ to 19.9‰ and 15.7‰ to 20.0‰, respectively, supporting the biogenic fidelity of these records. The δ^18^O values of two individuals from the nearby Sibrambang site (15.3–20.4‰, 14.7–20.8‰) are very similar to those of the Lida Ajer individual. These Sumatran fossils all fall at the upper end of the range of modern Sumatran orangutans (Figure 4), and reveal approximately annual δ^18^O periodicities (0.9–1.3 years), as well as strong bimodal distribution patterns in one instance (11565.162).

**Figure 4—figure supplement 1.**
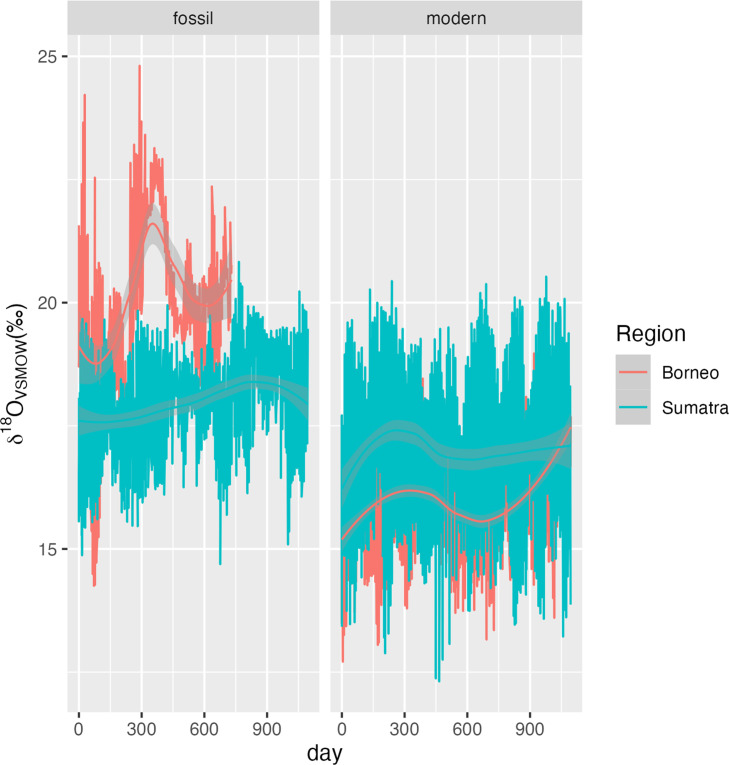
Comparison of δ^18^O values in fossil and modern orangutans from Borneo and Sumatra. Figure 4—figure supplement 1—source data 1.Numerical data used to generate this figure.

The two fossils from the Niah Caves were excavated from different regions and stratigraphic depths; δ^18^O values in the tooth from grid US/22 ranged from 15.9‰ to 24.8‰ and, unlike the three modern Bornean individuals, yielded an annual periodicity (1.0 years). The δ^18^O in the tooth from grid Y/F4 ranged from 14.2‰ to 22.9‰ and showed a stronger bimodal trend than an annual one, although its short formation time may have prohibited identification of longer trends. The range of values from these two fossil molars (14.2–24.8‰) markedly exceeds the range of modern Bornean orangutans (12.7–20.0‰) (Figure 4), with the mean δ^18^O value at least 2‰ heavier. This suggests possibly drier conditions with greater seasonality during fossil molar formation (Figure 4—figure supplement 1).

### Fossil orangutans—U-series age estimates

The six fossil teeth have very low uranium concentrations in their enamel (<0.5 ppm), regardless of their origin (Supplementary file 1). These enamel values are very close to the detection limit of the Nu Plasma II MC-ICP-MS, and thus are not useful for estimating minimum ages. The dentine of Lida Ajer specimen 11595.105 shows a spatial gradient of increasing uranium concentration from ~41 to 66 ppm, and decreasing age estimates from ~51 to 40 ka (Supplementary file 1). This trend might result from a preferential uranium leaching overprint near the end of the root. Spot DE10, positioned near the enamel-dentine junction (EDJ), is less likely to be impacted (Appendix 1—figure 3), and is thus assumed to provide the most reliable minimum age for the tooth, ~40 ka. Uranium values from Lida Ajer specimen 11594.12 show a similar trend of concentrations decreasing from ~31 to 24 ppm toward the root tip. However, the U-series age estimates remain constant within the range 31–34 ka across the dentine (Supplementary file 1; Appendix 1—figure 3). No evidence for a recent overprint is observed, supporting a minimum age of 33 ka. In summary, this individual’s age is at least 33 ka, and possibly >40 ka.

U-series analysis of the dentine of Sibrambang specimen 11565.162 shows a slight decreasing trend of uranium concentration from the EDJ to the root tip (from >60 ppm to <60 ppm), and corresponding increasing age estimates (56–62 ka) (Supplementary file 1; Appendix 1—figure 4). This might result from a slight uranium leaching overprint; a minimum age of 60 ka is likely for this tooth. The U-series age estimates obtained for Sibrambang specimen 11564.5 show a decreasing trend from the EDJ toward the circumpulpal dentine from 75 to 65 ka (Supplementary file 1; Appendix 1—figure 4). However, given the associated uncertainties, this trend might not be meaningful. An average dentine U-series age of 70.3±5.5 ka (2σ) may be regarded as a minimum age for the fossil, which is broadly consistent with the single age estimate obtained from the enamel (64 ka). In summary, the two teeth from Sibrambang yield U-series apparent ages of ~60–70 ka.

The uranium concentration measured across the dentine of the Niah Caves specimen from grid Y/F4 shows little variability, 4.2–4.9 ppm. The U-series age estimates are between 6.0 and 8.7 ka (Supplementary file 1; Appendix 1—figure 5). The average dentine U-series minimum age is 7.6±1.3 ka. Similarly, the Niah Cave specimen from grid US/22 shows a consistent uranium concentration through the dentine (1.3–1.4 ppm), with relatively large uncertainties that nonetheless bracket individual age estimates (Supplementary file 1; Appendix 1—figure 5). The average dentine U-series age is 8.8±3.0 ka. In summary, the two teeth from Niah Cave yield consistent apparent ages of ~8–9 ka, which should be regarded as a minimum age constraint for the fossils.

## Discussion

### Primate oxygen isotope compositions do not reveal a clear milk enrichment effect

Half of our modern sample, and potentially all of our fossil sample, are composed of M1s. These begin forming around birth and continue growing for 3 or more years (Smith, 2016). Orangutan infants rely exclusively on maternal milk during their first year of life, supplementing this with solid foods in the second year, which are increased until suckling ceases prior to 9 years of age (van Noordwijk et al., 2013; Smith et al., 2017). Our developmentally guided sampling approach allows us to examine fine-scaled trends in δ^18^O values during birth, exclusive nursing, supplemental feeding, and also after nursing ends (in those individuals with available serial molar teeth).

We find that five modern orangutans show only minor and inconsistently elevated δ^18^O values during the first year of life when compared to the subsequent year. These data do not support the hypothesis that primate infants have markedly elevated body water δ^18^O values during exclusive nursing. Data from the majority of 12 human M1s studied by Vaiglova et al., 2024, similarly reveal maximum δ^18^O values after the first year of tooth formation, well beyond the duration of exclusive milk intake. This is also evident in the M1 of a Neanderthal born in the spring (Smith et al., 2018a); δ^18^O values mostly rose for the first 3.5 months of life, but did not reach a maximum for another 2 years—long after the infant would have begun consuming supplemental foods and liquids. This final dataset points to the influence of season of birth on initial postnatal δ^18^O values, as inferred in other mammals (Bryant et al., 1996; Fricke and O’Neil, 1996).

Comparisons of serially forming teeth in four wild orangutans also fail to show a consistent elevation of δ^18^O values in M1s versus M2s (or M3s in two cases). Comparisons of M1 δ^18^O values with subsequent-forming teeth in four baboons, two tantalus monkeys, and one mona monkey (from Green et al., 2022: SI Dataset S1) also largely fail to support the enriched ‘Pattern 1’ trend modeled by Bryant et al., 1996: Figure 4, p. 401. This is also the case in comparisons of δ^18^O values from bulk samples of human teeth—Wright and Schwarcz, 1999, demonstrated that M1s have higher δ^18^O values than later-forming teeth in only four of seven individuals. In summary, the data from a range of primates including humans do not support the exclusion of early-forming primate teeth from the assessment of environmental seasonality.

### Modern orangutans show similar isotopic values across the islands of Borneo and Sumatra

The two Bornean juveniles from the Munich collection (ZSM 1981/48, ZSM 1981/87) reflect the environmental conditions of the late 1880s and early 1890s in Skalau—a region where orangutans might now be locally extinct. Similarly, the teeth from the two Sumatran individuals from the Munich collection (ZSM 1981/246, ZSM 1981/248) were collected prior to 1939 in northern Aceh, from where orangutans also have since disappeared (Spehar et al., 2018). While the individuals from northernmost Sumatra might have inhabited somewhat higher elevations than those from western Borneo, there does not appear to be an evident altitude effect (lower isotopic values at higher altitudes), as these four individuals show similar isotopic values, save for a single brief excursion below 12‰ in ZSM 1981/248 (Table 1, Figure 3). It is unknown to what extent local rainfall may have been isotopically distinct at the time the teeth were forming.

The δ^18^O values shown in Figure 1 reflect estimates of monthly and annual average precipitation from the Online Isotopes in Precipitation Calculator (3.0) compiled for https://www.waterisotopes.org. Actual measurements of precipitation δ^18^O from the islands of Borneo and Sumatra are extremely limited. The closest observation facilities to the ZSM orangutan locations yield similar patterns of modern annual rainfall δ^18^O variability (Belgaman et al., 2017), yet specific measurements from the six facilities that make up ‘Cluster 3’ in this reference are not available for comparison.

Other studies underscore the complexity of water transport in this region—multiple factors such as the oceanic origin of water vapor, cloud cover and type, and the post-condensation process influence the short-term variability of δ^18^O values in rainfall (Moerman et al., 2013; Suwarman et al., 2013; Belgaman et al., 2016). For example, Moerman et al., 2013, provided 5 years of daily rainfall δ^18^O measurements from Northern Borneo (Gunung Mulu National Park, Malaysia); daily rainfall δ^18^O values ranged from +0.7‰ to −18.5‰ and showed 1–3 month, annual, and supra-annual cycle frequencies. Interannual rainfall δ^18^O fluctuations of 6–8‰ were significantly correlated with ENSO events; these are similar in scale to the large fluctuations in our serial tooth datasets (Figure 3).

Another potential source of isotopic variability derives from dietary variation, as orangutans obtain the majority of their body water from plants (Mackinnon, 1974). Plant oxygen isotope compositions can be stratified within tropical forest canopies (da Silveira et al., 1989; Roberts et al., 2017; Lowry et al., 2021)—potentially leading to offset values among various animals, including primates, that consume different resources in the same forest (Krigbaum et al., 2013; Nelson, 2013; Fannin and Scott McGraw, 2020). Orangutans forage at different canopy heights ranging from the ground to high in the canopy (Mackinnon, 1974; Ungar, 1996; Thorpe and Crompton, 2005; Ashbury et al., 2015). Mackinnon, 1974, reported that Bornean and Sumatran orangutans obtain 95% of their food from the middle and upper levels of the canopy, where preferred foods are most abundant. In contrast, Ungar, 1996, reported that Sumatran orangutans were quite variable in feeding heights, with a mean of approximately 19 m; lower than gibbons who fed preferentially in the high canopy. Thorpe and Crompton, 2005, reported stratification in Sumatran orangutans, with immature individuals feeding below 20 m, females feeding both below and above this height, and adult/subadult males preferring to feed high in the canopy.

While differences in enamel δ^18^O values are apparent in comparisons of sympatric arboreal and terrestrial mammals (reviewed in Lowry et al., 2021; Green et al., 2022), it remains to be seen whether primates with broadly similar diets and habitats show meaningful differences in δ^18^O values, and to what degree plant physiology influences the pattern and amplitude of seasonality relative to rainfall. Oxygen isotope compositions in the six modern individuals from the islands of Borneo and Sumatra are very similar. Orangutans from both islands prefer ripe fruit when available, with some differences in the consumption of bark, leaves, unripe fruits, and insects—which varies between sites and across seasons (reviewed in Smith et al., 2012). Seasonal variation in diets and the stratification of food within the canopy may also contribute to enamel oxygen isotope variation within individuals, in addition to the seasonal rainfall trends we observe in our datasets. Orangutan δ^18^O values are also quite similar to the δ^18^O values from five humans from Flores, Indonesia (14.8–21.0‰) dated at ~2.2–3.0 ka (Vaiglova et al., 2024). This is remarkable given the major dietary differences between frugivorous orangutans and omnivorous coastal-dwelling humans, and suggests that their enamel δ^18^O values are predominantly influenced by regional precipitation.

### Fossil orangutan isotope values suggest different ancient climates in Sumatra and Borneo

Dating studies at Lida Ajer have established the presence of the oldest human remains in insular Southeast Asia, ~63–73 ka (Westaway et al., 2017), and a broad survey of the cave has reconfirmed an age of MIS 4 (59–76 ka) for the mammalian fauna (Louys et al., 2022). This is consistent with the minimum age of ~33–40 ka estimated for the two molars examined in the current study. The Sumatran Sibrambang Cave has been regarded as roughly contemporaneous to Lida Ajer given broad faunal similarities (de Vos, 1983). Recent U-series dating of two fossil orangutans from the Sibrambang assemblage yielded minimum ages of >56 ka and >85 ka (Louys et al., 2024), which bracket the apparent U-series minimum ages of ~60–70 ka in the current study. Sibrambang primates appear similar to, or slightly older than, those from Lida Ajer, given the minimum U-series age estimates for teeth from both sites, but this is not definitive given the absence of finite numerical ages for the fossils. Our analysis of δ^18^O values in Sumatran orangutan fossil molars reveals a close similarity across sites and with modern Sumatran individuals, although the fossil compositions fall at the upper end of the modern range. This may indicate a slightly dryer and less variable climate during the late Pleistocene; elevated tooth δ^18^O values are also indicative of elevated values in hydrological systems globally, resulting from increased ice volumes in glaciers and at the poles.

Pollen records from the Niah Caves archaeological site indicate that there were a number of local ecological shifts from lowland rainforest to more open environments during the Late Pleistocene and into the Holocene (Hunt et al., 2012), where humans may have begun hunting orangutans at ~45 ka (Spehar et al., 2018). While it is not possible to locate the two fossil orangutan molars in these pollen records, Piper and Rabett, 2016, considered that the large animal bone assemblages accumulated within the Lobang Hangus entrance and defined by the Harrisson spit depths of 12–42″ were of terminal Pleistocene age. More broadly, the orangutan specimen from grid US/22 (32–36″) is stratigraphically positioned between radiocarbon ages of 14,206–15,061 cal. BP (OxA-13936) and 36,583–38,059 cal. BP (OxA-13938), and this provides plausible minimum and maximum age constraints that are not incompatible with the apparent minimum U-series age of ~9 ka. Based on these results, the tooth is likely to date from the latest part of the Late Pleistocene. The specimen from grid Y/F4 might date from the latest part of the Late Pleistocene to the early Holocene, by comparison with the shell and fauna assemblage from other excavated areas (Piper et al., 2016.)

Both orangutan molars from the Niah Caves yield wide ranges of δ^18^O, which is particularly notable given the short periods of time sampled compared to the other fossils and most modern orangutan molars. Given the similar offsets in δ^18^O values between modern baboons living in Ugandan forests and the Ethiopian rift region (Green et al., 2022) and modern and prehistoric Bornean orangutans, we regard the higher δ^18^O values in the Niah Cave orangutans as possibly indicative of reduced rainfall when compared to recent conditions. This is consistent with paleoclimate reconstructions for Borneo and Flores during the late Pleistocene and early Holocene (Griffiths et al., 2009; Buckingham et al., 2022), when the environment around the Niah Caves is believed to have been a drier, more open seasonal forest (Harrison, 1996; Hunt et al., 2012). A study of δ^18^O values in Niah Caves shell middens dating from the early to mid-Holocene indicates a shift to periods of high rainfall with less variation than modern conditions (Stephens et al., 2016). The transition from a drier environment to moist tropical rainforest is also reflected in the increasing number and higher frequencies of canopy-adapted mammalian taxa in excavated layers of the Pleistocene-Holocene transition (Piper and Lim, 2021).

Our approach has the potential to contribute to reconstructions of ancient paleoenvironments in Southeast Asia based on studies of pollen, molluscs, faunal community compositions, guano records, and stable isotopes of teeth (e.g. Jablonski et al., 2000; Bird et al., 2005; Louys and Meijaard, 2010; Wurster et al., 2010; Hunt et al., 2012; Janssen et al., 2016; Stephens et al., 2016; Louys and Roberts, 2020; Bacon et al., 2021; Louys et al., 2022; Hamilton et al., 2024). This may be especially timely given that recent work examining modern fauna compositions in African landscapes has cautioned that fossil herbivore assemblages tend to overestimate the extent of ancient grasslands in comparison to woodlands (Negash and Barr, 2023; also see Sokolowski et al., 2023). Fine-scaled tooth sampling may also allow an expansion of inferences from δ^18^O values of bulk-sampled Asian hominin remains (Janssen et al., 2016; Roberts et al., 2020; Kubat et al., 2023), which are difficult to interpret for understanding seasonal rainfall dynamics in tropic environments (Green et al., 2022). Such information could better inform debates about whether humans employed arid savannah corridors to avoid dense tropical forests, or whether humans were adept at colonizing such environments during their consequential migration throughout island Southeast Asia.

## Materials and methods

### Orangutan samples

Thin (histological) sections of twelve molar teeth from six modern orangutans and six molar teeth from five fossil orangutans were employed (Table 1). These sections were previously prepared for studies of incremental tooth development, enamel thickness, elemental chemistry, and Asian hominoid taxonomy (Smith, 2016; Smith et al., 2011; Smith et al., 2012; Smith et al., 2017; Smith et al., 2018b). Four modern individuals were sourced from the Munich State Anthropological Collection (ZSM): two were collected in 1893–1894 from Skalau (north of the Kapuas River and south of the Klingkang Mountains in eastern West Borneo), and two were collected prior to 1939 from Aceh (northwest Sumatra) (Röhrer-Ertl, 1988: Figure 3, p. 14) (Figure 1). It was not possible to determine from which specific regions or time periods the two other modern individuals derive—collection notes were not available for these specimens from the Harvard Museum of Natural History (MCZ) or the Humboldt Museum (ZMB). Ages at death were determined for five of six individuals from assessments of incremental features and elemental registration of serially forming molars (detailed in Smith, 2016; Smith et al., 2017).

We also studied four Sumatran fossil orangutan teeth that were collected more than a century ago from the Lida Ajer and Sibrambang Caves in the Padang Highlands by Eugene Dubois (de Vos, 1983). Right and left lower molars from Lida Ajer (11594.12, 11595.105) show identical trace element patterns in their dentine (Appendix 1—figure 6), as well as similar occlusal fissure patterns and light wear, consistent with their attribution to the same individual. Two Bornean fossil orangutan teeth from Niah Caves (Malaysia) were also included in this study. The caves have yielded significant late Pleistocene and early Holocene human remains since the Harrissons began excavations in the 1950s (Barker et al., 2007). These lower molars were derived from two different entrances to the cave system, Gan Kira (grid square Y/F4) and Lobang Angus/Hangus (grid square US/22), with burial depths of 12–18″ and 30–36″, respectively (Hooijer, 1961). Although Hooijer, 1948, Hooijer, 1961, identified all six of these fossil teeth as M1s, we regard this as tentative, given that isolated orangutan molars are notoriously difficult to seriate (Grine and Franzen, 1994).

### Dating of fossil samples

Preliminary assessments at the Australian National University Radiocarbon Dating Laboratory confirmed that collagen preservation in the six fossil orangutans was insufficient for radiocarbon dating, a common limitation in tropical environments (e.g. Wood et al., 2016). Laser ablation uranium series (U-series) analyses were carried out on longitudinal sections of teeth at the Radiogenic Isotope Facility of the University of Queensland using an ASI RESOlution SE laser ablation system connected to a Nu Plasma II MC-ICP-MS. A succession of several rasters (<2 min linear ablations) was made in a transect across the dentine and enamel of each tooth (Appendix 1—figures 3–5) following Grün et al., 2014. The ^230^Th/^238^U and ^234^U/^238^U activity ratios of the samples were normalized to bracketing analyses of a homogeneous rhino tooth standard that has been precisely calibrated by isotope dilution (Grün et al., 2014). Importantly, dental tissues are known to behave as open systems for U-series elements; provided there is no occurrence of uranium leaching, age estimates should therefore be regarded as minimum age constraints since uranium uptake into dental tissues may be significantly delayed after death.

### Tooth formation and oxygen isotope analyses

Thin sections were first imaged with transmitted light microscopy. Enamel daily secretion rates were measured between sequential accentuated growth lines to yield the time of formation (see Smith, 2016: Figure 1, p. 94), and enamel extension rates were calculated between accentuated lines to guide placement of the analyzed spots at approximately weekly intervals of growth from the dentine horn tip to the enamel cervix (Smith et al., 2018a; Green et al., 2022). Following the removal of cover slips by immersion in xylene, each thin section was analyzed for δ^18^O at the SHRIMP Laboratory at the Australian National University according to methods detailed in Vaiglova et al., 2024.

In brief, a 15 kV Cs primary ion beam focused to a spot ~15 × 20 µm diameter was used to sequentially sample the enamel as close as possible to the EDJ. Oxygen secondary ions were extracted at 10 kV and analyzed isotopically by a multiple collector equipped with dual electrometers operated in resistor mode. The δ^18^O values were calculated relative to reference apatite (Durango 3) measured every 10–15 sample analyses. Distances of SHRIMP δ^18^O measurements along the innermost enamel from the cusp to cervix were converted to secretory time in days following Green et al., 2022. A polynomial regression relating distances to days was created using the enamel extension rates, and this regression was applied to estimate the timing of secretory deposition at every SHRIMP spot location. The Lomb-Scargle periodogram was used to assess time-dependent patterns of δ^18^O values, which estimates the power of sine wave periods within a given range to produce the temporal patterns present within those measurements.

The probability that differences between first and second year δ^18^O values in modern first molars might have arisen by chance was assessed by one-way paired t-tests, with alpha = 0.05 adjusted by Bonferroni correction due to repeated comparisons across multiple teeth. Figure and data plotting using Python 3 in the Google Colab environment were aided by ChatGPT, a language model based on the GPT-3.5 architecture developed by OpenAI.

## Figures and Tables

**Appendix 1—figure 1.**
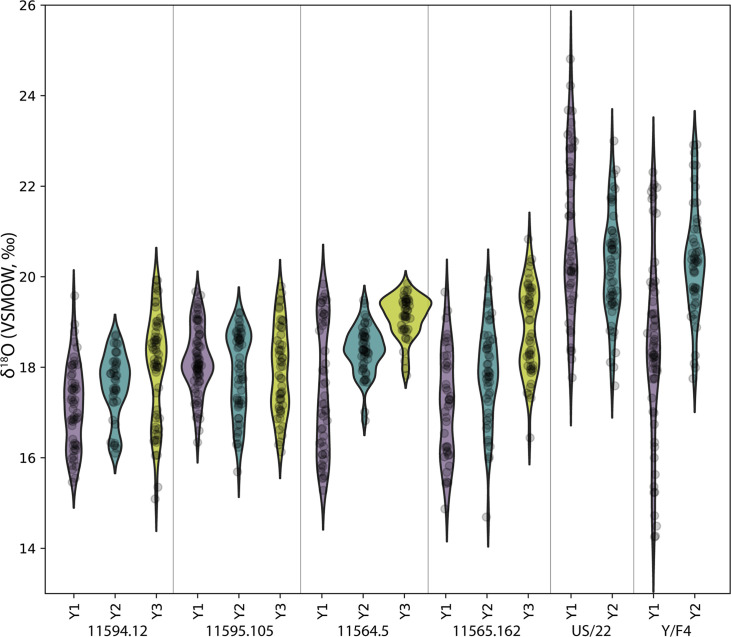
Comparison of sequential δ^18^O values across multiple years of molar formation in six putative fossil orangutan M1s from Borneo and Sumatra. The width of each curve is a kernel density estimate (KDE) corresponding to the distribution of oxygen isotope values measured from different teeth. From each tooth, first year data (Y1) is shown with a purple violin plot, second year data (Y2) with a green plot, and third year data (Y3, if present) with a yellow plot. Actual data are plotted as black circles.

**Appendix 1—figure 2.**
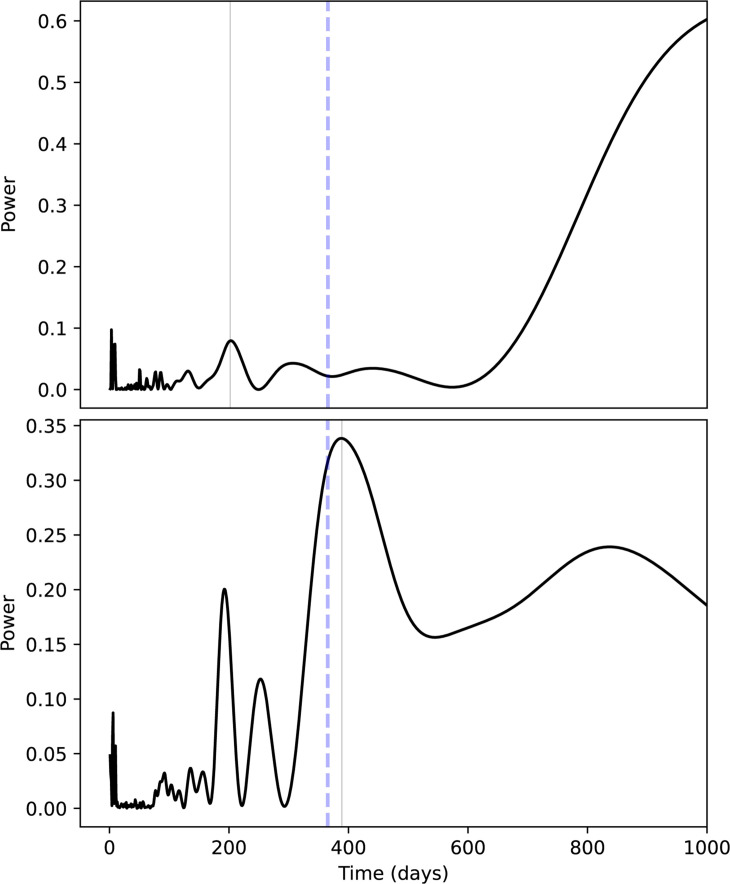
Inferred seasonality of δ^18^O values from a Bornean (top) and Sumatran (bottom) M1. Lomb-Scargle periodograms show potential periods in days (x-axis) against period power (y-axis), where higher values on the y-axis indicate underlying sine wave periods that produce, contribute to, or explain δ^18^O value oscillations. Best-fit periodicities are shown as light gray vertical lines, whereas annual periodicities are indicated by blue dashed horizontal lines. We regard the Bornean M1 (MCZ 5290) as largely aperiodic; a minor peak is observed at c. 6 months, and increasing powers at very high periods are an artifact of limited sampling length within teeth relative to the model. The Sumatran M1 (ZMB 83508) has a 1.1 year inferred frequency, as well as an approximately 6-month peak, likely reflecting semiannual monsoons.

**Appendix 1—figure 3.**
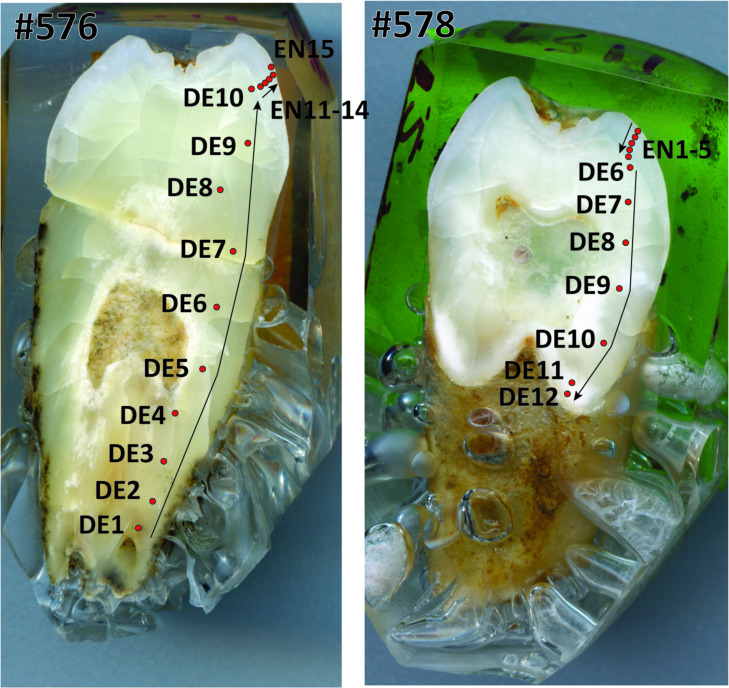
Laser ablation profiles performed across the two teeth from Lida Ajer. #576 (left) refers to specimen 11595.105; #578 (right) refers to specimen 11594.12. The red dots represent the position of the rasters, and arrows indicate the sequence of the analyses. EN = enamel. DE = dentine.

**Appendix 1—figure 4.**
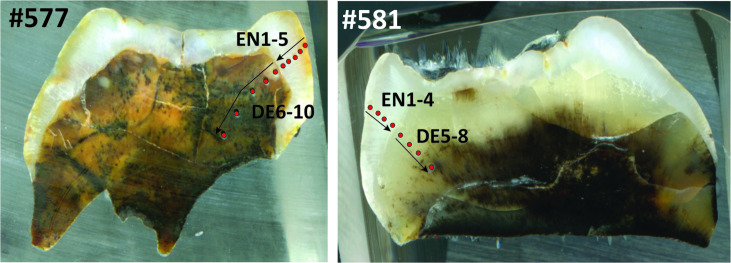
Laser ablation profiles across the two teeth from Sibrambang Cave. #577 (left) refers to specimen 11565.162; #581 (right) refers to specimen 11564.5. The red dots represent the position of the rasters, and arrows indicate the sequence of the analyses. EN = enamel. DE = dentine.

**Appendix 1—figure 5.**
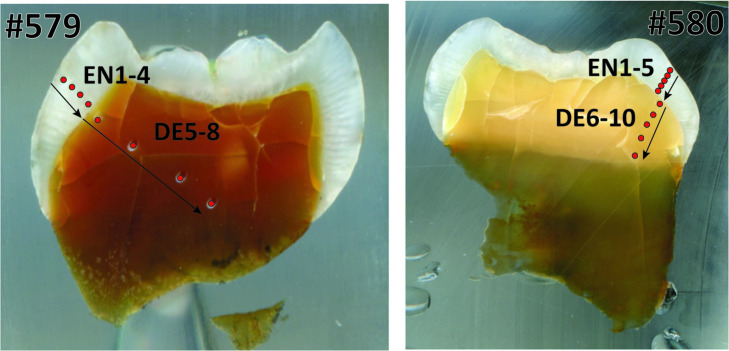
Laser ablation profiles across the two teeth from Niah Caves. #579 (left) refers to specimen from grid Y/F4; #580 (right) refers to specimen from grid US/22. The red dots represent the position of the rasters, and arrows indicate the sequence of the analyses. EN = enamel. DE = dentine.

**Appendix 1—figure 6.**
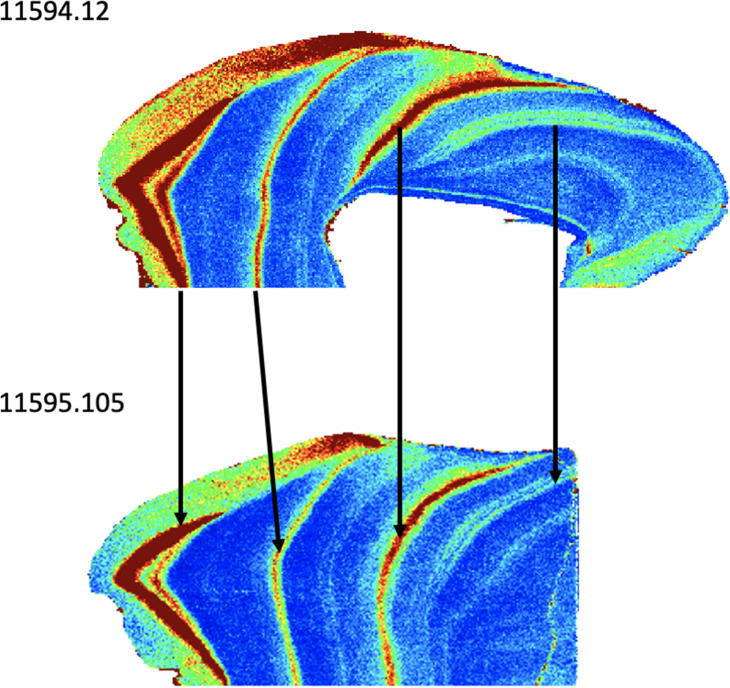
Matching trace element patterns in cross-sections of two isolated molars from the Dubois collection of fossil orangutan teeth from Lida Ajer. High concentrations are shown in warm colors, low concentration are in cool colors; here Li/Ca is shown, but identical corresponding patterns were also observed for Ba/Ca and Sr/Ca (not shown). The enamel cap of each tooth is to the left, and root dentine is to the right. Trace elements were measured according to LA-ICP-MS methods detailed in Smith et al., 2017.

## Data Availability

All data generated or analysed during this study are included in the manuscript and supporting files; source data files have been provided for Figures 2–4, Figure 4—figure supplement 1, and Appendix 1—figure 1.

## Additional files

Supplementary file 1. U-series dates for fossil orangutan material. ^230^Th/^238^U and ^234^U/^238^U are activity ratios. It is worth noting that, for most transect analyses, the ^232^Th signal, which was measured on a Faraday collector, was indistinguishable from background noise. In this regard, the corresponding ^230^Th/^232^Th activity ratio of each transect should be >>100, and thus non-radiogenic or detrital ^230^Th correction would have negligible impact on the age. U-series data in italics should be viewed with caution due to U concentrations of ≤0.5 ppm. All errors are 2-σ. Key: EN = enamel; DE = dentine; n.c.=not calculable. Negative values were caused by background extraction from their measured peaks with intensities at detection levels.

MDAR checklist

Source data 1. Source data for Figures 2—4, Appendix 1—figures 1 and 2.

## References

[bib1] Aldrian E, Dwi Susanto R (2003). Identification of three dominant rainfall regions within Indonesia and their relationship to sea surface temperature. International Journal of Climatology.

[bib2] Ao H, Ruan J, Martinón-Torres M, Krapp M, Liebrand D, Dekkers MJ, Caley T, Jonell TN, Zhu Z, Huang C, Li X, Zhang Z, Sun Q, Yang P, Jiang J, Li X, Xie X, Song Y, Qiang X, Zhang P, An Z (2024). Concurrent Asian monsoon strengthening and early modern human dispersal to East Asia during the last interglacial. PNAS.

[bib3] Archibald S, Bond WJ, Hoffmann W, Lehmann C, Staver C, Stevens N, Scogings PF, Sankaran M (2019). Savanna Woody Plants and Large Herbivores.

[bib4] Ashbury AM, Posa MRC, Dunkel LP, Spillmann B, Atmoko SSU, van Schaik CP, van Noordwijk MA (2015). Why do orangutans leave the trees? Terrestrial behavior among wild Bornean orangutans (Pongo pygmaeus wurmbii) at Tuanan, Central Kalimantan. American Journal of Primatology.

[bib5] Aubert M, Williams IS, Boljkovac K, Moffat I, Moncel MH, Dufour E, Grün R (2012). In situ oxygen isotope micro-analysis of faunal material and human teeth using A SHRIMP II: A new tool for palaeo-ecology and archaeology. Journal of Archaeological Science.

[bib6] Bacon A-M, Bourgon N, Welker F, Cappellini E, Fiorillo D, Tombret O, Thi Mai Huong N, Anh Tuan N, Sayavonkhamdy T, Souksavatdy V, Sichanthongtip P, Antoine P-O, Duringer P, Ponche J-L, Westaway K, Joannes-Boyau R, Boesch Q, Suzzoni E, Frangeul S, Patole-Edoumba E, Zachwieja A, Shackelford L, Demeter F, Hublin J-J, Dufour É (2021). A multi-proxy approach to exploring *Homo sapiens*’ arrival, environments and adaptations in Southeast Asia. Scientific Reports.

[bib7] Bae CJ, Douka K, Petraglia MD (2017). On the origin of modern humans: Asian perspectives. Science.

[bib8] Barker G, Barton H, Bird M, Daly P, Datan I, Dykes A, Farr L, Gilbertson D, Harrisson B, Hunt C, Higham T, Kealhofer L, Krigbaum J, Lewis H, McLaren S, Paz V, Pike A, Piper P, Pyatt B, Rabett R, Reynolds T, Rose J, Rushworth G, Stephens M, Stringer C, Thompson J, Turney C (2007). The “human revolution” in lowland tropical Southeast Asia: the antiquity and behavior of anatomically modern humans at Niah Cave (Sarawak, Borneo). Journal of Human Evolution.

[bib9] Belgaman HA, Ichiyanagi K, Tanoue M, Suwarman R (2016). Observational research on stable isotopes in precipitation over indonesian maritime continent. Journal of Japanese Association of Hydrological Sciences.

[bib10] Belgaman HA, Ichiyanagi K, Suwarman R, Tanoue M, Aldrian E, Utami AID, Kusumaningtyas SDA (2017). Characteristics of seasonal precipitation isotope variability in Indonesia. Hydrological Research Letters.

[bib11] Bird MI, Taylor D, Hunt C (2005). Palaeoenvironments of insular Southeast Asia during the last glacial period: a savanna corridor in Sundaland?. Quaternary Science Reviews.

[bib12] Blumenthal SA, Levin NE, Brown FH, Brugal JP, Chritz KL, Harris JM, Jehle GE, Cerling TE (2017). Aridity and hominin environments. PNAS.

[bib13] Bowen GJ (2010). Isoscapes: spatial pattern in isotopic biogeochemistry. Annual Review of Earth and Planetary Sciences.

[bib14] Britton K, Fuller BT, Tütken T, Mays S, Richards MP (2015). Oxygen isotope analysis of human bone phosphate evidences weaning age in archaeological populations. American Journal of Physical Anthropology.

[bib15] Brockman D, Van Schaik C (2005). Seasonality in Primates: Studies of Living and Extinct Human and Non-Human Primates (Cambridge Studies in Biological and Evolutionary Anthropology).

[bib16] Bryant JD, Froelich PN, Showers WJ, Genna BJ (1996). A Tale of two quarries: biologic and taphonomic signatures in the oxygen isotope composition of tooth enamel phosphate from modern and miocene equids. PALAIOS.

[bib17] Buckingham FL, Carolin SA, Partin JW, Adkins JF, Cobb KM, Day CC, Ding Q, He C, Liu Z, Otto‐Bliesner B, Roberts WHG, Lejau S, Malang J (2022). Termination 1 millennial‐scale rainfall events over the sunda shelf. Geophysical Research Letters.

[bib18] Cherney MD, Fisher DC, Hren MT, Shirley EA (2021). Stable isotope records of nursing and weaning: a case study in elephants with implications for paleobiological investigations. Palaeogeography, Palaeoclimatology, Palaeoecology.

[bib19] Chesson LA, Valenzuela LO, O’Grady SP, Cerling TE, Ehleringer JR (2010). Hydrogen and oxygen stable isotope ratios of milk in the United States. Journal of Agricultural and Food Chemistry.

[bib20] Cole JE, Fairbanks RG, Shen GT (1993). Recent variability in the southern oscillation: isotopic results from a tarawa atoll coral. Science.

[bib21] Curran LM, Caniago I, Paoli GD, Astianti D, Kusneti M, Leighton M, Nirarita CE, Haeruman H (1999). Impact of El Nino and logging on canopy tree recruitment in borneo. Science.

[bib22] Dansgaard W (1964). Stable isotopes in precipitation. Tellus.

[bib23] da Silveira L, Sternberg L, Mulkey SS, Joseph Wright S (1989). Oxygen isotope ratio stratification in a tropical moist forest. Oecologia.

[bib24] de Vos J (1983). The Pongo faunas from Java and Sumatra and their significance for biostratigraphical and paleo-ecological interpretations. Palaeontology Proceedings B.

[bib25] Fannin LD, Scott McGraw W (2020). Does Oxygen stable isotope composition in primates vary as a function of vertical stratification or folivorous behaviour?. Folia Primatologica.

[bib26] Fricke HC, O’Neil JR (1996). Inter- and intra-tooth variation in the oxygen isotope composition of mammalian tooth enamel phosphate: implications for palaeoclimatological and palaeobiological research. Palaeogeography, Palaeoclimatology, Palaeoecology.

[bib27] Green DR, Olack G, Colman AS (2018). Determinants of blood water *δ*
^18^O variation in a population of experimental sheep: implications for paleoclimate reconstruction. Chemical Geology.

[bib28] Green DR, Ávila JN, Cote S, Dirks W, Lee D, Poulsen CJ, Williams IS, Smith TM (2022). Fine-scaled climate variation in equatorial Africa revealed by modern and fossil primate teeth. PNAS.

[bib29] Griffiths ML, Drysdale RN, Gagan MK, Zhao J -X, Ayliffe LK, Hellstrom JC, Hantoro WS, Frisia S, Feng Y -X, Cartwright I, Pierre ESt, Fischer MJ, Suwargadi BW (2009). Increasing Australian–Indonesian monsoon rainfall linked to early Holocene sea-level rise. Nature Geoscience.

[bib30] Grine F, Franzen JL (1994). Fossil hominid teeth from the Sangiran Dome (Java, Indonesia). Courier Forschungsinstitut Senckenberg.

[bib31] Grün R, Eggins S, Kinsley L, Moseley H, Sambridge M (2014). Laser ablation U-series analysis of fossil bones and teeth. Palaeogeography, Palaeoclimatology, Palaeoecology.

[bib32] Hamilton R, Amano N, Bradshaw CJA, Saltré F, Patalano R, Penny D, Stevenson J, Wolfhagen J, Roberts P (2024). Forest mosaics, not savanna corridors, dominated in Southeast Asia during the last glacial maximum. PNAS.

[bib33] Harrison T (1996). The paleoecological context at Niah Cave Sarawak: evidence from the primate fauna. Bulletin of the Indo-Pacific Prehistory Association.

[bib34] Hooijer DA (1948). Prehistoric teeth of man and of the orang-utan from central Sumatra, with notes on the fossil orang-utan from Java and Southern China. Zoologische Mededelingen.

[bib35] Hooijer DA (1961). The orang-utan in Niah Cave prehistory. Sarawak Museum Journal.

[bib36] Hughen KA, Schrag DP, Jacobsen SB, Hantoro W (1999). El Niño during the Last Interglacial Period recorded by a fossil coral from Indonesia. Geophysical Research Letters.

[bib37] Hunt CO, Gilbertson DD, Rushworth G (2012). A 50,000-year record of late Pleistocene tropical vegetation and human impact in lowland Borneo. Quaternary Science Reviews.

[bib38] Jablonski NG, Whitfort MJ, Roberts-Smith N, Qinqi X (2000). The influence of life history and diet on the distribution of catarrhine primates during the Pleistocene in eastern Asia. Journal of Human Evolution.

[bib39] Janssen R, Joordens JCA, Koutamanis DS, Puspaningrum MR, de Vos J, van der Lubbe JHJL, Reijmer JJG, Hampe O, Vonhof HB (2016). Tooth enamel stable isotopes of Holocene and Pleistocene fossil fauna reveal glacial and interglacial paleoenvironments of hominins in Indonesia. Quaternary Science Reviews.

[bib40] Knott CD (1998). Changes in orangutan caloric intake, energy balance, and ketones in response to fluctuating fruit availability. International Journal of Primatology.

[bib41] Kornexl BE, Werner T, Roßmann A, Schmidt HL (1997). Measurement of stable isotope abundances in milk and milk ingredients -- A possible tool for origin assignment and quality control. Zeitschrift Fur Lebensmitteluntersuchung Und -Forschung A.

[bib42] Krigbaum J, Berger MH, Daegling DJ, McGraw WS (2013). Stable isotope canopy effects for sympatric monkeys at Tai Forest, Cote d’Ivoire. Biology Letters.

[bib43] Kubat J, Nava A, Bondioli L, Dean MC, Zanolli C, Bourgon N, Bacon AM, Demeter F, Peripoli B, Albert R, Lüdecke T, Hertler C, Mahoney P, Kullmer O, Schrenk F, Müller W (2023). Dietary strategies of Pleistocene Pongo sp. and Homo erectus on Java (Indonesia). Nature Ecology & Evolution.

[bib44] Lin GP, Rau YH, Chen YF, Chou CC, Fu WG (2006). Measurements of δD and δ^18^O stable isotope ratios in milk. Journal of Food Science.

[bib45] Louys J, Meijaard E (2010). Palaeoecology of Southeast Asian megafauna‐bearing sites from the Pleistocene and a review of environmental changes in the region. Journal of Biogeography.

[bib46] Louys J, Roberts P (2020). Environmental drivers of megafauna and hominin extinction in Southeast Asia. Nature.

[bib47] Louys J, Duval M, Price GJ, Westaway K, Zaim Y, Rizal Y, Puspaningrum M, Trihascaryo A, Breitenbach SFM, Kwiecien O, Cai Y, Higgins P, Albers PCH, de Vos J, Roberts P, Aswan (2022). Speleological and environmental history of Lida Ajer cave, western Sumatra. Philosophical Transactions of the Royal Society B.

[bib48] Louys J, Price GJ, Higgins P, de Vos J, Zaim J, Rizal Y, Aswan P, Tri Hascaryo MR, Drawhorn A, Louys J, Albers PCH, van der AAE (2024). Quaternary Palaeontology and Archaeology of Sumatra.

[bib49] Lowry BE, Wittig RM, Pittermann J, Oelze VM (2021). Stratigraphy of stable isotope ratios and leaf structure within an African rainforest canopy with implications for primate isotope ecology. Scientific Reports.

[bib50] Luyt J, Sealy J (2018). Inter-tooth comparison of δ13C and δ18O in ungulate tooth enamel from south-western Africa. Quaternary International.

[bib51] Mackinnon J (1974). The behaviour and ecology of wild orang-utans (Pongo pygmaeus). Animal Behaviour.

[bib52] Marshall AJ, Ancrenaz M, Brearley FQ, Fredriksson GM, Ghaffar N, Heydon M, Husson SJ, Leighton M, McConkey KR, Morrogh-Bernard HC, Proctor J, van CP, Yeager CP, Wich SA, Marshall AJ (2009). Orangutans: Geographic Variation in Behavioral Ecology and Conservation.

[bib53] Moerman JW, Cobb KM, Adkins JF, Sodemann H, Clark B, Tuen AA (2013). Diurnal to interannual rainfall δ18O variations in northern Borneo driven by regional hydrology. Earth and Planetary Science Letters.

[bib54] Moron V, Robertson AW, Boer R (2009). Spatial coherence and seasonal predictability of monsoon onset over Indonesia. Journal of Climate.

[bib55] Negash EW, Barr WA (2023). Relative abundance of grazing and browsing herbivores is not a direct reflection of vegetation structure: Implications for hominin paleoenvironmental reconstruction. Journal of Human Evolution.

[bib56] Nelson SV (2013). Chimpanzee fauna isotopes provide new interpretations of fossil ape and hominin ecologies. Proceedings. Biological Sciences.

[bib57] Pederzani S, Britton K (2019). Oxygen isotopes in bioarchaeology: Principles and applications, challenges and opportunities. Earth-Science Reviews.

[bib58] Piper PJ, Oxenham M, Buckley HR (2016). The Routledge Handbook of Bioarchaeology in Southeast Asia and the Pacific Islands.

[bib59] Piper P, Lewis H, Reynolds T, McLaren S, Rabett R, Cole F, Szábo K, Farr L, Barker G, Kira Gan, Hitam Kain, Farr L (2016). Archaeological Investigations in the Niah Caves, Sarawak: The Archaeology of the Niah Caves, Sarawak.

[bib60] Piper P, Rabett R, Barker G, Farr L (2016). Archaeological Investigations in the Niah Caves, Sarawak: The Archaeology of the Niah Caves, Sarawak.

[bib61] Piper PJ, Lim TT (2021). Zooarchaeology at Niah Cave: contributions to our understanding of southeast Asian prehistory. Malayan Nature Journal.

[bib62] Pletcher E, Staver C, Schwartz NB (2022). The environmental drivers of tree cover and forest–savanna mosaics in Southeast Asia. Ecography.

[bib63] Prendergast AL, Pryor AJE, Reade H, Stevens RE (2018). Seasonal records of palaeoenvironmental change and resource use from archaeological assemblages. Journal of Archaeological Science.

[bib64] Pumijumnong N, Bräuning A, Sano M, Nakatsuka T, Muangsong C, Buajan S (2020). A 338-year tree-ring oxygen isotope record from Thai teak captures the variations in the Asian summer monsoon system. Scientific Reports.

[bib65] Qian JH, Robertson AW, Moron V (2013). Diurnal cycle in different weather regimes and rainfall variability over borneo associated with ENSO. Journal of Climate.

[bib66] Roberts SB, Coward WA, Ewing G, Savage J, Cole TJ, Lucas A (1988). Effect of weaning on accuracy of doubly labeled water method in infants. American Journal of Physiology-Regulatory, Integrative and Comparative Physiology.

[bib67] Roberts P, Blumenthal SA, Dittus W, Wedage O, Lee-Thorp JA (2017). Stable carbon, oxygen, and nitrogen, isotope analysis of plants from a South Asian tropical forest: implications for primatology. American Journal of Primatology.

[bib68] Roberts P, Louys J, Zech J, Shipton C, Kealy S, Carro SS, Hawkins S, Boulanger C, Marzo S, Fiedler B, Boivin N, Aplin K, OʼConnor S (2020). Isotopic evidence for initial coastal colonization and subsequent diversification in the human occupation of Wallacea. Nature Communications.

[bib69] Röhrer-Ertl O, Schwartz JH (1988). Orang-utan Biology.

[bib70] Rozanski K, Araguás-Araguás L, Gonfiantini R, Swart PK, Lohmann KC, Mckenzie J, Savin S (1993). Climate Change in Continental Isotopic Records.

[bib71] Smith TM, Bacon A-M, Demeter F, Kullmer O, Nguyen KT, De Vos J, Wei W, Zermeno JP, Zhao L (2011). Dental tissue proportions in fossil orangutans from mainland Asia and Indonesia. Human Origins Research.

[bib72] Smith TM, Kupczik K, Machanda Z, Skinner MM, Zermeno JP (2012). Enamel thickness in Bornean and Sumatran orangutan dentitions. American Journal of Physical Anthropology.

[bib73] Smith TM (2016). Dental development in living and fossil orangutans. Journal of Human Evolution.

[bib74] Smith TM, Austin C, Hinde K, Vogel ER, Arora M (2017). Cyclical nursing patterns in wild orangutans. Science Advances.

[bib75] Smith TM, Austin C, Green DR, Joannes-Boyau R, Bailey S, Dumitriu D, Fallon S, Grün R, James HF, Moncel MH, Williams IS, Wood R, Arora M (2018a). Wintertime stress, nursing, and lead exposure in Neanderthal children. Science Advances.

[bib76] Smith TM, Houssaye A, Kullmer O, Le Cabec A, Olejniczak AJ, Schrenk F, de Vos J, Tafforeau P (2018b). Disentangling isolated dental remains of Asian Pleistocene hominins and pongines. PLOS ONE.

[bib77] Smith TM, Austin C, Ávila JN, Dirks W, Green DR, Williams IS, Arora M (2022). Permanent signatures of birth and nursing initiation are chemically recorded in teeth. Journal of Archaeological Science.

[bib78] Sokolowski KG, Codding BF, Du A, Faith JT (2023). Do grazers equal grasslands? Strengthening paleoenvironmental inferences through analysis of present-day African mammals. Palaeogeography, Palaeoclimatology, Palaeoecology.

[bib79] Spehar SN, Sheil D, Harrison T, Louys J, Ancrenaz M, Marshall AJ, Wich SA, Bruford MW, Meijaard E (2018). Orangutans venture out of the rainforest and into the Anthropocene. Science Advances.

[bib80] Stahle DW, Cleaveland MK, Therrell MD, Gay DA, D’Arrigo RD, Krusic PJ, Cook ER, Allan RJ, Cole JE, Dunbar RB, Moore MD, Stokes MA, Burns BT, Villanueva-Diaz J, Thompson LG (1998). Experimental dendroclimatic reconstruction of the southern oscillation. Bulletin of the American Meteorological Society.

[bib81] Stephens M, Rose J, Mattey D, Gilbertson D. D, Barker G, Farr L (2016). Archaeological Investigations in the Niah Caves: The Archaeology of the Niah Caves, Sarawak.

[bib82] Suwarman R, Ichiyanagi K, Tanoue M, Yoshimura K, Mori S, Yamanaka MD, Kurita N, Syamsudin F (2013). The variability of stable isotopes and water origin of precipitation over the maritime continent. SOLA.

[bib83] Thorpe SKS, Crompton RH (2005). Locomotor ecology of wild orangutans (Pongo pygmaeus abelii) in the Gunung Leuser Ecosystem, Sumatra, Indonesia: a multivariate analysis using log-linear modelling. American Journal of Physical Anthropology.

[bib84] Thorpe SKS, Crompton RH, Wich SA, Utami Atmoko SS, Setia TM, van CP (2009). Orangutans: Geographic Variation in Behavioral Ecology and Conservation.

[bib85] Tshen LT (2016). Biogeographic distribution and metric dental variation of fossil and living orangutans (Pongo spp.). Primates; Journal of Primatology.

[bib86] Tudhope AW, Chilcott CP, McCulloch MT, Cook ER, Chappell J, Ellam RM, Lea DW, Lough JM, Shimmield GB (2001). Variability in the El Niño-Southern Oscillation through a glacial-interglacial cycle. Science.

[bib87] Ungar PS (1996). Feeding Height and niche separation in sympatric sumatran Monkeys and Apes. Folia Primatologica.

[bib88] Urban FE, Cole JE, Overpeck JT (2000). Influence of mean climate change on climate variability from a 155-year tropical Pacific coral record. Nature.

[bib89] Vaiglova P, Ávila JN, Buckley H, Galipaud JC, Green DR, Halcrow S, James HF, Kinaston R, Oxenham M, Paz V, Simanjuntak T, Snoeck C, Trinh HH, Williams IS, Smith TM (2024). Past rainfall patterns in Southeast Asia revealed by microanalysis of δ18O values in human teeth. Journal of Archaeological Science.

[bib90] van Noordwijk MA, Willems EP, Utami Atmoko SS, Kuzawa CW, van Schaik CP (2013). Multi-year lactation and its consequences in Bornean orangutans (Pongo pygmaeus wurmbii). Behavioral Ecology and Sociobiology.

[bib91] Van Schaik CP (1986). Phenological changes in a Sumatran rain forest. Journal of Tropical Ecology.

[bib92] Vico G, Dralle D, Feng X, Thompson S, Manzoni S (2017). How competitive is drought deciduousness in tropical forests? A combined eco-hydrological and eco-evolutionary approach. Environmental Research Letters.

[bib93] Wessling EG, Kühl HS, Mundry R, Deschner T, Pruetz JD (2018). The costs of living at the edge: seasonal stress in wild savanna-dwelling chimpanzees. Journal of Human Evolution.

[bib94] Westaway KE, Louys J, Awe RD, Morwood MJ, Price GJ, Zhao J-X, Aubert M, Joannes-Boyau R, Smith TM, Skinner MM, Compton T, Bailey RM, van den Bergh GD, de Vos J, Pike AWG, Stringer C, Saptomo EW, Rizal Y, Zaim J, Santoso WD, Trihascaryo A, Kinsley L, Sulistyanto B (2017). An early modern human presence in Sumatra 73,000-63,000 years ago. Nature.

[bib95] Wood R, Duval M, Mai Huong NT, Tuan NA, Bacon AM, Demeter F, Duringer P, Oxenham M, Piper P (2016). The effect of grain size on carbonate contaminant removal from tooth enamel: Towards an improved pretreatment for radiocarbon dating. Quaternary Geochronology.

[bib96] Wright LE, Schwarcz HP (1999). Correspondence between stable carbon, oxygen and nitrogen isotopes in human tooth enamel and dentine: infant diets at Kaminaljuyú. Journal of Archaeological Science.

[bib97] Wurster CM, Bird MI, Bull ID, Creed F, Bryant C, Dungait JAJ, Paz V (2010). Forest contraction in north equatorial Southeast Asia during the Last Glacial Period. PNAS.

[bib98] Yang H, Johnson KR, Griffiths ML, Yoshimura K (2016). Interannual controls on oxygen isotope variability in Asian monsoon precipitation and implications for paleoclimate reconstructions. Journal of Geophysical Research.

