# Supplementary material for: Oxygen isotopes in orangutan teeth reveal recent and ancient climate variation
Source: eLife. 2024 Mar 8;12:RP90217. doi: 10.7554/eLife.90217 (PMC10942278; doi:10.7554/eLife.90217)
Supplement: Supplementary file 1. — 230Th/238U and 234U/238U are activity ratios. It is worth noting that, for most transect analyses, the 232Th signal, which was measured on a Faraday collector, was indistinguishable from background noise. In this regard, the corresponding 230Th/232Th activity ratio of each transect should be >>100, and thus non-radiogenic or detrital 230Th correction would have negligible impact on the age. U-series data in italics should be viewed with caution due to U concentrations of ≤0.5 ppm. All errors are 2-σ. Key: EN = enamel; DE = dentine; n.c.=not calculable. Negative values were caused by background extraction from their measured peaks with intensities at detection levels. [file elife-90217-supp1.docx]

Supplementary File 2. U-series dates for fossil orangutan material.

| **LA raster** | **Tissue** | **U(ppm)** | **^230^Th/^238^U** | **±** | **^234^U/^238^U** | **±** | **Age (ka)** | **±** |
| --- | --- | --- | --- | --- | --- | --- | --- | --- |
| 11595.105-EN11 | enamel | *0.02* | *-1.230* | *0.090* | *0.459* | *0.037* | n.c. | n.c. |
| 11595.105-EN12 | enamel | *0.00* | *-12.82* | *0.084* | *-4.590* | *0.018* | n.c. | n.c. |
| 11595.105-EN13 | enamel | *0.00* | *-31.04* | *0.082* | *-17.11* | *0.018* | n.c. | n.c. |
| 11595.105-EN14 | enamel | *0.10* | *0.623* | *0.168* | *1.155* | *0.105* | n.c. | n.c. |
| 11595.105-EN15 | enamel | *0.04* | *-0.826* | *0.082* | *0.752* | *0.051* | n.c. | n.c. |
| 11595.105-DE1 | dentine | *41.4* | *0.516* | *0.020* | *1.360* | *0.047* | 50.6 | 3.40 |
| 11595.105-DE2 | dentine | *44.2* | *0.470* | *0.020* | *1.359* | *0.055* | 45.2 | 3.33 |
| 11595.105-DE3 | dentine | *48.6* | *0.471* | *0.015* | *1.344* | *0.045* | 46.0 | 2.65 |
| 11595.105-DE4 | dentine | *58.9* | *0.481* | *0.015* | *1.356* | *0.038* | 46.6 | 2.50 |
| 11595.105-DE5 | dentine | *61.1* | *0.482* | *0.019* | *1.341* | *0.045* | 47.5 | 3.10 |
| 11595.105-DE6 | dentine | *65.8* | *0.446* | *0.019* | *1.346* | *0.051* | 43.0 | 3.04 |
| 11595.105-DE7 | dentine | *64.0* | *0.440* | *0.016* | *1.346* | *0.044* | 42.3 | 2.55 |
| 11595.105-DE8 | dentine | *60.0* | *0.430* | *0.016* | *1.338* | *0.046* | 41.4 | 2.56 |
| 11595.105-DE9 | dentine | *64.6* | *0.433* | *0.018* | *1.336* | *0.049* | 41.8 | 2.81 |
| 11595.105-DE10 | dentine | *65.9* | *0.411* | *0.016* | *1.325* | *0.047* | 39.7 | 2.53 |

| **LA raster** | **Tissue** | **U(ppm)** | **^230^Th/^238^U** | **±** | **^234^U/^238^U** | **±** | **Age (ka)** | **±** |
| --- | --- | --- | --- | --- | --- | --- | --- | --- |
| 11594.12-EN1 | enamel | *0.00* | *-13.08* | *0.082* | *-9.394* | *0.019* | n.c. | n.c. |
| 11594.12-EN2 | enamel | *0.00* | *-23.91* | *0.104* | *-11.05* | *0.016* | n.c. | n.c. |
| 11594.12-EN3 | enamel | *0.00* | *-22.35* | *0.078* | *-4.621* | *0.027* | n.c. | n.c. |
| 11594.12-EN4 | enamel | *0.00* | *-16.49* | *0.089* | *-6.110* | *0.015* | n.c. | n.c. |
| 11594.12-EN5 | enamel | *0.01* | *17.58* | *0.103* | *16.21* | *0.031* | *224* | *3.12* |
| 11594.12-DE6 | dentine | 30.9 | 0.337 | 0.016 | 1.335 | 0.041 | 31.2 | 2.02 |
| 11594.12-DE7 | dentine | 30.6 | 0.347 | 0.013 | 1.337 | 0.036 | 32.2 | 1.68 |
| 11594.12-DE8 | dentine | 31.9 | 0.355 | 0.017 | 1.332 | 0.049 | 33.3 | 2.31 |
| 11594.12-DE9 | dentine | 31.4 | 0.366 | 0.013 | 1.335 | 0.035 | 34.4 | 1.78 |
| 11594.12-DE10 | dentine | 32.1 | 0.359 | 0.011 | 1.329 | 0.038 | 33.8 | 1.71 |
| 11594.12-DE11 | dentine | 24.6 | 0.344 | 0.014 | 1.357 | 0.031 | 31.4 | 1.67 |
| 11594.12-DE12 | dentine | 24.9 | 0.352 | 0.015 | 1.351 | 0.032 | 32.3 | 1.85 |

| **LA raster** | **tissue** | **U(ppm)** | **^230^Th/^238^U** | **±** | **^234^U/^238^U** | **±** | **Age (ka)** | **±** |
| --- | --- | --- | --- | --- | --- | --- | --- | --- |
| 11565.162-EN1 | enamel | 0.58 | 0.434 | 2.630 | 1.025 | 1.495 | 59.7 | 496 |
| 11565.162-EN2 | enamel | *0.00* | *-4.899* | *0.132* | *-81.42* | *0.020* | n.c. | n.c. |
| 11565.162-EN3 | enamel | *0.00* | *-23.35* | *0.075* | *-16.84* | *0.018* | n.c. | n.c. |
| 11565.162-EN4 | enamel | *0.00* | *-37.09* | *0.082* | *-22.74* | *0.021* | n.c. | n.c. |
| 11565.162-EN5 | enamel | *0.01* | *-5.283* | *0.068* | *-0.022* | *0.029* | n.c. | n.c. |
| 11565.162-DE6 | dentine | 62.2 | 0.438 | 0.287 | 1.073 | 0.470 | 56.5 | 59.4 |
| 11565.162-DE7 | dentine | 63.6 | 0.461 | 0.023 | 1.066 | 0.048 | 61.2 | 5.73 |
| 11565.162-DE8 | dentine | 61.4 | 0.467 | 0.021 | 1.072 | 0.044 | 61.7 | 5.23 |
| 11565.162-DE9 | dentine | 59.8 | 0.470 | 0.028 | 1.073 | 0.060 | 62.2 | 7.01 |
| 11565.162-DE10 | dentine | 57.4 | 0.480 | 0.026 | 1.072 | 0.051 | 64.2 | 6.51 |

| **LA raster** | **tissue** | **U(ppm)** | **^230^Th/^238^U** | **±** | **^234^U/^238^U** | **±** | **Age (ka)** | **±** |
| --- | --- | --- | --- | --- | --- | --- | --- | --- |
| 11564.5-EN1 | enamel | 0.11 | 0.304 | 0.181 | 0.959 | 0.086 | 41.6 | 30.6 |
| 11564.5-EN2 | enamel | *0.00* | *-2215* | *0.086* | *-486.53* | *0.018* | n.c. | n.c. |
| 11564.5-EN3 | enamel | *0.00* | *-97.95* | *0.065* | *-4.236* | *0.031* | n.c. | n.c. |
| 11564.5-EN4 | enamel | 9.86 | 0.502 | 0.211 | 1.115 | 0.162 | 64.3 | 38.8 |
| 11564.5-DE5 | dentine | 51.6 | 0.564 | 0.055 | 1.116 | 0.052 | 75.4 | 11.8 |
| 11564.5-DE6 | dentine | 45.8 | 0.541 | 0.057 | 1.111 | 0.047 | 71.6 | 11.6 |
| 11564.5-DE7 | dentine | 49.1 | 0.530 | 0.039 | 1.117 | 0.079 | 69.0 | 10.2 |
| 11564.5-DE8 | dentine | 47.8 | 0.512 | 0.010 | 1.128 | 0.016 | 65.0 | 2.13 |

| **LA raster** | **tissue** | **U(ppm)** | **^230^Th/^238^U** | **±** | **^234^U/^238^U** | **±** | **Age (ka)** | **±** |
| --- | --- | --- | --- | --- | --- | --- | --- | --- |
| Y/F4-EN1 | enamel | *0.01* | *-4.737* | *0.084* | *-0.283* | *0.025* | n.c. | n.c. |
| Y/F4-EN2 | enamel | *0.00* | *-42.98* | *0.070* | *-2.119* | *0.024* | n.c. | n.c. |
| Y/F4-EN3 | enamel | *0.00* | *-137.8* | *0.078* | *-18.50* | *0.017* | n.c. | n.c. |
| Y/F4-EN4 | enamel | *0.00* | *-49.66* | *0.079* | *3.398* | *0.033* | n.c. | n.c. |
| Y/F4-DE5 | dentine | 4.41 | 0.058 | 0.016 | 1.082 | 0.031 | 5.97 | 1.74 |
| Y/F4-DE6 | dentine | 4.93 | 0.072 | 0.018 | 1.057 | 0.034 | 7.69 | 1.99 |
| Y/F4-DE7 | dentine | 4.80 | 0.077 | 0.018 | 1.088 | 0.031 | 7.95 | 1.93 |
| Y/F4-DE8 | dentine | 4.15 | 0.085 | 0.041 | 1.102 | 0.065 | 8.74 | 4.43 |

| **LA raster** | **tissue** | **U(ppm)** | **^230^Th/^238^U** | **±** | **^234^U/^238^U** | **±** | **Age (ka)** | **±** |
| --- | --- | --- | --- | --- | --- | --- | --- | --- |
| US/22-EN1 | enamel | *0.00* | *-7.442* | *0.102* | *-6.047* | *0.023* | n.c. | n.c. |
| US/22-EN2 | enamel | *0.00* | *-53.99* | *0.082* | *-14.98* | *0.020* | n.c. | n.c. |
| US/22-EN3 | enamel | *0.00* | *215.0* | *0.103* | *78.83* | *0.019* | n.c. | n.c. |
| US/22-EN4 | enamel | *0.00* | *2.054* | *0.109* | *-12.69* | *0.020* | n.c. | n.c. |
| US/22-EN5 | enamel | *0.02* | *-2.955* | *0.109* | *1.044* | *0.052* | n.c. | n.c. |
| US/22-DE6 | dentine | 1.28 | 0.066 | 0.070 | 1.205 | 0.100 | 6.09 | 6.66 |
| US/22-DE7 | dentine | 1.36 | 0.085 | 0.073 | 1.179 | 0.086 | 8.10 | 7.22 |
| US/22-DE8 | dentine | 1.38 | 0.094 | 0.060 | 1.183 | 0.071 | 8.94 | 5.98 |
| US/22-DE9 | dentine | 1.41 | 0.126 | 0.062 | 1.183 | 0.087 | 12.27 | 6.38 |
| US/22-DE10 | dentine | 1.41 | 0.090 | 0.050 | 1.234 | 0.080 | 8.17 | 4.77 |

^230^Th/^238^U and ^234^U/^238^U are activity ratios. It is worth noting that, for most transect analyses, the ^232^Th signal, which was measured on a Faraday collector, was indistinguishable from background noise. In this regard, the corresponding ^230^Th/^232^Th activity ratio of each transect should be >>100, and thus non-radiogenic or detrital ^230^Th correction would have negligible impact on the age. U-series data in italics should be viewed with caution due to U concentrations of ≤ 0.5 ppm. All errors are 2-σ. Key: EN= enamel; DE = dentine; n.c. = not calculable. Negative values were caused by background extraction from their measured peaks with intensities at detection levels.
